# GOLD grade-specific characterization of COPD in the COSYCONET multi-center trial: comparison of semiquantitative MRI and quantitative CT

**DOI:** 10.1007/s00330-024-11269-3

**Published:** 2025-01-08

**Authors:** Philip Konietzke, Oliver Weinheimer, Simon M. F. Triphan, Sebastian Nauck, Felix Wuennemann, Marilisa Konietzke, Bertram J. Jobst, Rudolf A. Jörres, Claus F. Vogelmeier, Claus P. Heussel, Hans-Ulrich Kauczor, Mark O. Wielpütz, Jürgen Biederer, on behalf of the COSYCONET study group

**Affiliations:** 1 Department of Diagnostic and Interventional Radiology, University Hospital of Heidelberg, Heidelberg, Germany; 2 Translational Lung Research Center Heidelberg (TLRC), German Center for Lung Research (DZL), University of Heidelberg, Heidelberg, Germany; 3 Department of Diagnostic and Interventional Radiology with Nuclear Medicine, Thoraxklinik at University of Heidelberg, Heidelberg, Germany; 4 Institute and Outpatient Clinic for Occupational, Social and Environmental Medicine, Ludwig-Maximilians-University, Munich, Germany; 5 Comprehensive Pneumology Center Munich (CPC-M), Munich, Germany; 6 Department of Medicine, Pulmonary and Critical Care Medicine, University Medical Center Giessen and Marburg, German Center for Lung Research (DZL), Marburg, Germany; 7 Diagnostic Radiology and Neuroradiology, Greifswald University Hospital, Ferdinand-Sauerbruch-Strasse 1, Greifswald, Germany; 8 Faculty of Medicine, University of Latvia, Riga, Latvia; 9 Faculty of Medicine, Christian-Albrechts-Universität zu Kiel, Kiel, Germany

**Keywords:** Magnetic resonance imaging, Computed tomography, Chronic obstructive pulmonary disease, Pulmonary emphysema

## Abstract

**Objectives:**

We hypothesized that semiquantitative visual scoring of lung MRI is suitable for GOLD-grade specific characterization of parenchymal and airway disease in COPD and that MRI scores correlate with quantitative CT (QCT) and pulmonary function test (PFT) parameters.

**Methods:**

Five hundred ninety-eight subjects from the COSYCONET study (median age = 67 (60–72)) at risk for COPD or with GOLD1-4 underwent PFT, same-day paired inspiratory/expiratory CT, and structural and contrast-enhanced MRI. QCT assessed total lung volume (TLV), emphysema, and air trapping by parametric response mapping (PRM_Emph_, PRM_fSAD_) and airway disease by wall percentage (WP). MRI was analyzed using a semiquantitative visual scoring system for parenchymal defects, perfusion defects, and airway abnormalities. Descriptive statistics, Spearman correlations, and ANOVA analyses were performed.

**Results:**

TLV, PRM_Emph_, and MRI scores for parenchymal and perfusion defects were all higher with each GOLD grade, reflecting the extension of emphysema (all *p* < 0.001). Airway analysis showed the same trends with higher WP and higher MRI large airway disease scores in GOLD3 and lower WP and MRI scores in GOLD4 (*p* = 0.236 and *p* < 0.001). Regional heterogeneity was less evident on MRI, while PRM_Emph_ and MRI perfusion defect scores were higher in the upper lobes, and WP and MRI large airway disease scores were higher in the lower lobes. MRI parenchymal and perfusion scores correlated moderately with PRM_Emph_ (*r* = 0.61 and *r* = 0.60) and moderately with FEV1/FVC (*r* = −0.56).

**Conclusion:**

Multi-center semiquantitative MRI assessments of parenchymal and airway disease in COPD matched GOLD grade-specific imaging features on QCT and detected regional disease heterogeneity. MRI parenchymal disease scores were correlated with QCT and lung function parameters.

**Key Points:**

***Question*** *Do MRI-based scores correlate with QCT and PFT parameters for GOLD-grade specific disease characterization of COPD*?

***Findings*** *MRI can visualize the parenchymal and airway disease features of COPD*.

***Clinical relevance*** *Lung MRI is suitable for GOLD-grade specific disease characterization of COPD and may serve as a radiation-free imaging modality in scientific and clinical settings, given careful consideration of its potential and limitations*.

## Introduction

Chronic obstructive pulmonary disease (COPD) is a heterogeneous disease characterized by varying contributions of emphysema and airway abnormalities to lung function impairment. The diagnosis is based on symptoms and spirometry, and airflow limitation is commonly categorized according to the global initiative for obstructive lung disease (GOLD) criteria [1].

Quantitative computed tomography (QCT) provides an accurate assessment of the severity and distribution of emphysema and airway disease, two imaging key features of COPD [2–4]. Emphysema can be assessed by lung density analysis using established indices such as the emphysema index (EI) [5, 6]. Quantification of airway disease is more challenging, as small airways (< 2 mm inner diameter), which are the primary sites of airflow restriction in COPD, lie below the resolution limit of clinical CT scanners [7, 8]. For the indirect assessment of small airway disease (SAD), “air trapping” can be identified on expiratory CT scans [9, 10]. However, the use of fixed threshold measurements to quantify “air trapping” may not discriminate between emphysema and SAD. Therefore, parametric response mapping (PRM) has been proposed, an integrated approach that uses ex- and inspiratory CT scans to classify each voxel as normal lung, emphysema, or functional SAD (fSAD) [9]. QCT has already been used in several large-scale multi-center studies such as ECLIPSE and COPDGene for COPD monitoring and phenotyping [11, 12]. Nevertheless, CT is associated with non-negligible radiation exposure, which can add up considerably over repeated CT examinations [13].

Magnetic resonance imaging (MRI) is an alternative, radiation-free modality that avoids cumulative radiation exposure and enables the evaluation of morphological and functional changes in the lungs. Lung MRI is already been established in patients with cystic fibrosis [14–17] and has shown promising results in the assessment of COPD in single-center studies [18, 19]. Recently, a COSYCONET substudy showed considerable agreement between semiquantitative MRI and CT scores in the visual phenotyping of COPD [20].

Therefore, we hypothesized (1) that semiquantitative MRI may be suitable for GOLD grade-specific characterization of emphysema and airway disease in COPD, (2) that semiquantitative MRI scores correlate with QCT parameters, and (3) that semiquantitative MRI scores correlate with lung function parameters.

## Materials and methods

### Study design

The study was approved by the Institutional Review Boards of all participating study centers and by the German Federal Office for Radiation Protection, and all subjects provided written informed consent prior to the study. The trial (trial registration: German Clinical Trials Register DRKS00005072) was embedded into the German “Impact of Systemic Manifestations/Comorbidities on Clinical State, Prognosis, Utilization of Health Care Resources in Patients with COPD” study (COSYCONET, Clinicaltrials.gov identifier NCT01245933), substudy: “Image-Based Structural and Functional Phenotyping of the COSYCONET Cohort Using MRI and CT” (MR-COPD, NCT02629432). COSYCONET is a prospective multi-center study that has enrolled more than 2700 subjects [21].

**Fig. 1:**
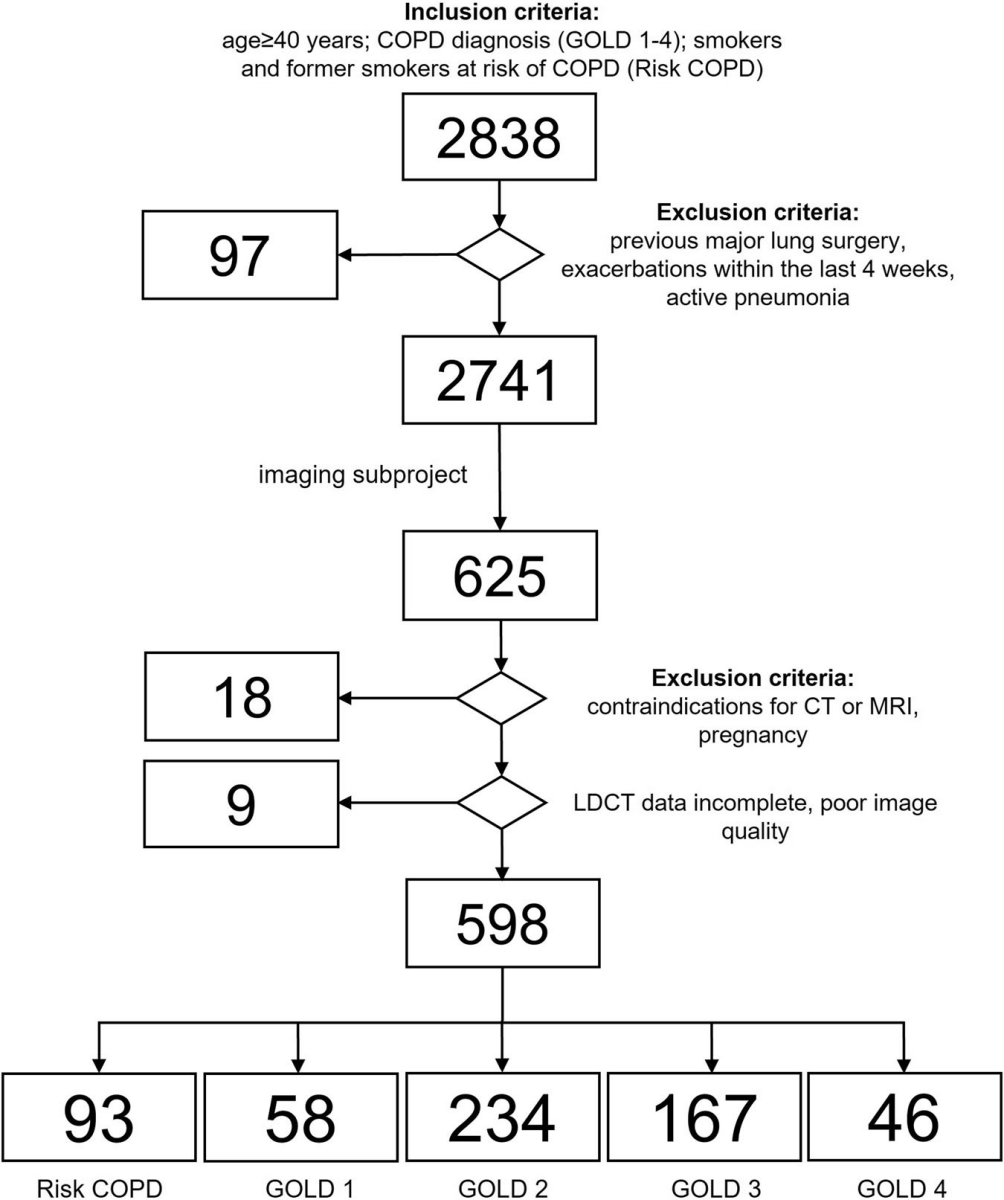
Patient recruitment flowchart

The present substudy was designed to evaluate the suitability of MRI for GOLD grade-specific characterization of emphysema and airway disease in COPD and to assess the agreement of semiquantitative MRI scores with QCT and pulmonary function test (PFT) parameters. Therefore, 598 subjects from the COSYCONET cohort were enrolled at 15 COSYCONET study centers over a period of 3 years, and detailed inclusion and exclusion criteria can be found in the literature [21]. Additional exclusion criteria for the imaging substudy are provided in the recruitment flowchart (Fig. 1).

All subjects were diagnosed with COPD grade 1–4 according to the GOLD consortium, based on a ratio of forced expiratory pressure in 1 s to forced vital capacity (FEV1/FVC) < 0.7 [1]. In addition, smokers and ex-smokers without an assigned GOLD category, specifically those in the former “GOLD0 category” were included and subsumed as “risk COPD”. This group included individuals with FEV1/FVC ≥ 0.7, but with a physician-based diagnosis of COPD.

### Lung function assessments

All subjects underwent whole-body plethysmography, spirometry, and the assessment of lung diffusing capacity within 63 ± 146 days of image acquisition, performed and analyzed according to the American Thoracic Society and European Respiratory Society recommendations [22]. In this study, we focused on the ratio of FEV1/FVC (Table 1).

**Table 1:** Patient demographics and body plethysmography parameters

	All GOLD grades	Risk COPD	GOLD1	GOLD2	GOLD3	GOLD4	*p*
Demographics							
*n*	598	93	58	234	167	46	<−
Age [y]	67 (60–72)	67 (59–72)	68 (61–75)	67 (61–72)	66 (59–71)	63 (55–69)	> 0.006
Sex [f/m]	233/365	41/52	19/39	142/92	65/102	30/16	<−
BMI [kg/m²]	26 (23–30)	29 (26–32)	27 (23–30)	27 (24–30)	26 (23–29)	23 (21–26)	< 0.001
PYs [y]	37 (16–62)	34 (10–58)	44 (24–63)	40 (19–64)	35 (14–58)	37 (19–65)	> 0.105
Lung function parameters							
TLC [L]	7.2 (6.1–8.2)	5.9 (5.2–7.3)	7.6 (6.4–8.3)^*^	7.3 (6.2–8.2)	7.3 (6.3–8.3)	8.2 (7.0–9.0)	< 0.001
VC [L]	3.3 (2.6–4.1)	3.3 (2.5–4.1)	4.1 (3.6–4.9)^*^	3.6 (2.9–4.2)^*^	3.0 (2.4-3.6)^*^	2.3 (1.9-2.8)^*^	< 0.001
FVC [L]	3.1 (2.4–3.8)	3.1 (2.3–3.8)	4.1 (3.6–4.9)^*^	3.4 (2.7–3.9)^*^	2.8 (2.2–3.3)^*^	2.0 (1.8–2.4)^*^	< 0.001
RV [L]	3.6 (2.9–4.4)	2.7 (2.2–3.2)	3.0 (2.6–3.6)	3.5 (3.0–4.2)^*^	4.2 (3.5–5.0)^*^	5.4 (4.4–6.3)^*^	< 0.001
FRC [L]	4.6 (3.7–5.5)	3.2 (2.9–3.8)	4.0 (3.7–4.9)^*^	4.6 (3.9–5.3)	5.2 (4.4–6.0)^*^	6.5 (5.4–7.2)^*^	< 0.001
FEV1 [L]	1.7 (1.2–2.2)	2.3 (1.8–3.0)	2.6 (2.3–2.9)	1.8 (1.5–2.1)^*^	1.2 (1.0–1.4)^*^	0.8 (0.7–0.9)^*^	< 0.001
FEV1/FVC [%]	55 (45–65)	76 (72–80)	63 (60–66)^*^	56 (49–62)^*^	45 (38–51)^*^	36 (33–43)^*^	< 0.001
T_LCO_^#^	4.8 (3.6–6.3)	5.9 (4.9–7.5)	6.2 (4.7–7.0)	5.0 (3.9–6.6)	4.0 (3.0–4.9)^*^	2.8 (1.9–3.9)	< 0.001
Lung function parameters predicted							
FVCpp [%]	91 (61–89)	91 (78–104)	114 (106–122)^*^	96 (86–106)^*^	78 (68–92)^*^	55 (49–68)^*^	< 0.001
FEV1pp [%]	57 (44–75)	82 (73–92)	86 (83–91)	62 (55–69)^*^	42 (37–46)^*^	25 (21–29)^*^	< 0.001

Patient demographics (age, sex, pack-years (PYs) and BMI and lung function parameters comprising total lung capacity (TLC), vital capacity (VC), FVC, RV, functional residual capacity (FRC), FEV1, ratio of FEV1/FVC, FVC in percent predicted (FVCpp), FEV1 in percent predicted (FEV1pp), and transfer factor of the lung for carbon monoxide (T_LCO_)). Results are shown for all patients and the different GOLD grades (risk COPD, GOLD1, GOLD2, GOLD3, and GOLD4). All data are given as median with interquartile range (Q1–Q3)

^*^ *p* < 0.001 vs previous GOLD grade

^#^ mmol/min/kPa

### CT and MRI image acquisition

MRI and CT examinations were performed on the same day. CT examinations were performed on clinical CT scanners of different manufacturers with at least 40-row detector arrays. The standardized non-enhanced CT protocol employed inspiratory and end-expiratory spiral acquisitions of the total lung in thin collimation (Supplemental Table 1). MRI examinations were performed using clinical 1.5-T or 3.0-T MRI scanners according to a standardized chest MRI protocol (Supplemental Table 2).

### Quantitative post-processing of CT images

The validated scientific software (YACTA v2.8.2.3) segmented the lungs and the individual lobes fully automatically on the inspiratory and expiratory images as previously described [23–27].

Emphysema was assessed by the total lung volume (TLV), EI, and mean lung density (MLD). PRM was performed after deformable CT volume registration, which allows the combination of inspiratory and expiratory CT lung scans to classify individual lung parenchyma voxels as normal (PRM_Normal_), voxels with functional small airways disease (PRM_fSAD_) and emphysema (PRM_Emph_) by assuming that lung voxels with inspiratory CT attenuation less than −950 Hounsfield units (HU) represent emphysema, while voxels with values greater than −950 HU on inspiration but less than −856 HU on expiration represent fSAD [9, 28]. All variables were computed for the total lung and all lobes separately (i.e., right upper (RUL), middle (RML), and lower (RLL) lobe, as well as left upper lobe (LUL), lingula (LLi) and left lower lobe (LLL) and for the combined upper lung regions (ULR = RUL + RML + LUL + LLi) and lower lung regions (LLR = RLL + LLL) lung regions.

The bronchiectasis index (BE) was calculated for the total lung. The other airway parameters were analyzed using an airway generation- and lobe-based approach. Wall thickness (WT), total diameter (TD), lumen area (LA), and wall percentage (WP) were calculated for each generation (generation 1–10) and the results were combined for central (airway generation 1–2 (G_1–2_)), large = lobar and segmental (airway generation 3–5 (G_3–5_)), and subsegmental (airway generation (G_6–10_)) airways. The lobe-based approach calculated the BE and WP for all individual lobes, and the results for the combined upper lung region (ULR) and LLR were pooled. Further details on the quantitative post-processing can be found in the online supplementary material.

### Semiquantitative MRI assessment

MR images were visually evaluated using OsiriX software on a dedicated workstation with two 21” certified medical image monitors. Two radiologists with 3 years of experience in lung imaging analyzed the images independently. Both studies from each patient were read separately by each reader, who was blinded to the images and results from the other modality. A minimum of 2 weeks was allowed between readings of MRI and CT to minimize recall bias. Finally, the records of the two first readers were reviewed by a third reader with more than 20 years of experience in pulmonary MRI as an adjudicator to reach a consensus.

**Fig. 2:**
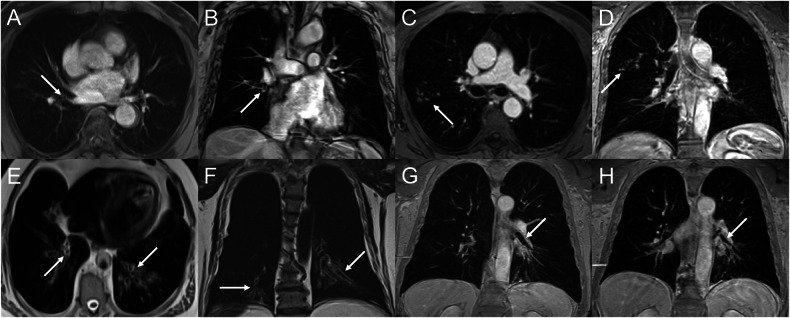
MIR airway pathologies. **A**, **B** Transversal and coronal post-contrast VIBE images showing airway wall thickening of the right lower lobe bronchi (white arrows). **C**, **D** Transversal and coronal post-contrast VIBE images showing a tree-in-bud sign in the right upper lobe (white arrows). **E**, **F** Transversal and coronal T2 HASTE images showing bronchiectasis and associated wall thickening in both lower lobes (white arrows). **G**, **H** Coronal post-contrast VIBE images during inspiration (**G**) and expiration (**H**) with the expiratory collapse of the left main bronchus (white arrows)

Semiquantitative visual scoring of COPD-related pathologies was performed, using a previously established MRI scoring system in cystic fibrosis and COPD [14, 29]. Lung parenchymal defects as an indicator of emphysema and lung perfusion defects as an indicator of functional disease (small airways disease and emphysema) were scored on a 3-point scale for all six lobes (0 = absent, 1 = ≤ 50%, 2 = > 50% of lobe affected). Multiple MRI features of central, large, and SAD were rated binary (0 = not present, 1 = present) or using a 3-point-scale for each lobe (0 = absent, 1 = ≤ 50%, 2 = > 50% of the airways affected) (Fig. 2). Central airway disease (wall thickening/expiratory collapse) was scored binary in the trachea and right and left main bronchi. Large airway disease (bronchiectasis/wall thickening) was scored on a 3-point scale per lobe and expiratory collapse of the lobar bronchi was scored binary. SAD (tree-in-bud appearance and peripheral bronchiectasis (PBE)) were scored on a 3-point scale per lobe. Finally, an airway score (central + large + small airways score) and a global disease score (functional disease + airway score) were calculated for the total lung (Supplemental Table 3). Additional information and inter-reader agreements for MRI subscores are available in the online supplement (Supplemental Table 4). In addition, a detailed discussion of the MRI subscores can be found in a previously published article [20].

### Statistical analyses

Statistical analyses were performed using R (R4.3.1, Foundation for Statistical Computing) and SigmaPlot14.0 (Systat Software GmbH). Demographics, PFT parameters, quantitative CT parameters, and semiquantitative MRI scores are reported as median with interquartile range (Q1–Q3). Data with assumed skewed distributions were analyzed using the Mann–Whitney test to compare two unmatched groups (lung region comparison) or the Kruskal–Wallis test to compare three or more unmatched groups (GOLD grade comparison). Dunn’s test was used for multiple pairwise comparisons. A *p*-value < 0.05 was considered statistically significant. Spearman rank correlation was calculated between semiquantitative MRI, QCT, and PFT and interpreted as follows: 0.00–0.10 = negligible, 0.10–0.39 = weak, 0.40–0.69 = moderate, 0.70–0.89 = strong, and 0.90–1.00= very strong [30]. Inter-reader variability for MRI scores was assessed using Fleiss kappa, with agreement levels of 0–0.20 = poor, 0.21–0.40 = fair, 0.41–0.60 =moderate, 0.61–0.80 = substantial, and 0.81–1.00 = almost perfect [31].

## Results

### Patient population

As part of the COSYCONET study, in total 598 patients (297 women, 301 men, aged 65.6 ± 8.3 years) at risk for COPD or with GOLD grades 1–4 were recruited (Fig. 1 and Table 1).

### GOLD grade-specific quantitative CT

**Fig. 3:**
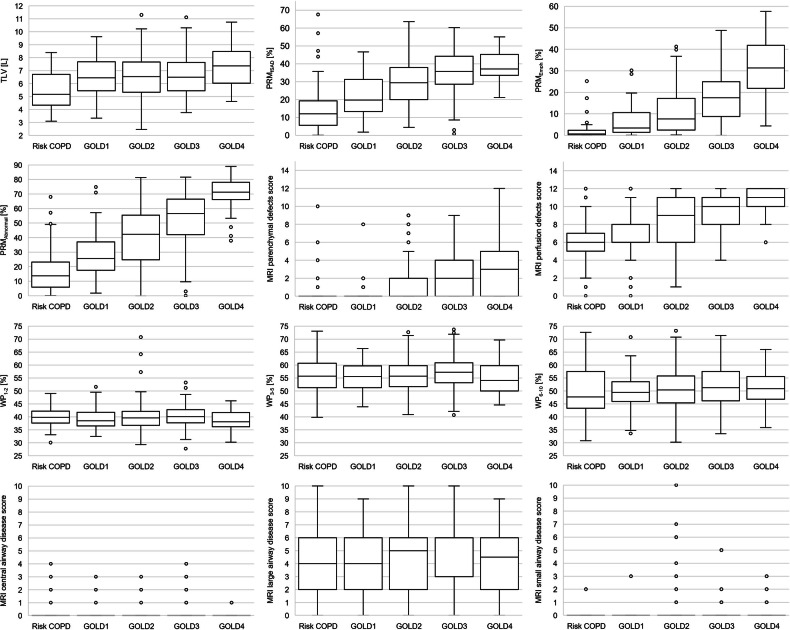
Box plots for quantitative CT parameters. Box plots of the parenchymal disease parameters TLV, PRM (PRM_fSAD_, PRM_Emph_, and PRM_Abnormal_), MRI parenchymal and perfusion defect scores, and the airway disease parameters WP (WP_1–2_, WP_3–5_, and WP_6–10_) and MRI central, large and SAD scores for the COPD stages risk COPD–GOLD1–4. Boxes indicate the 25–75th percentile, whiskers indicate the 5th and 95th percentile, and individual outliers are indicated by white-filled circles

**Fig. 4:**
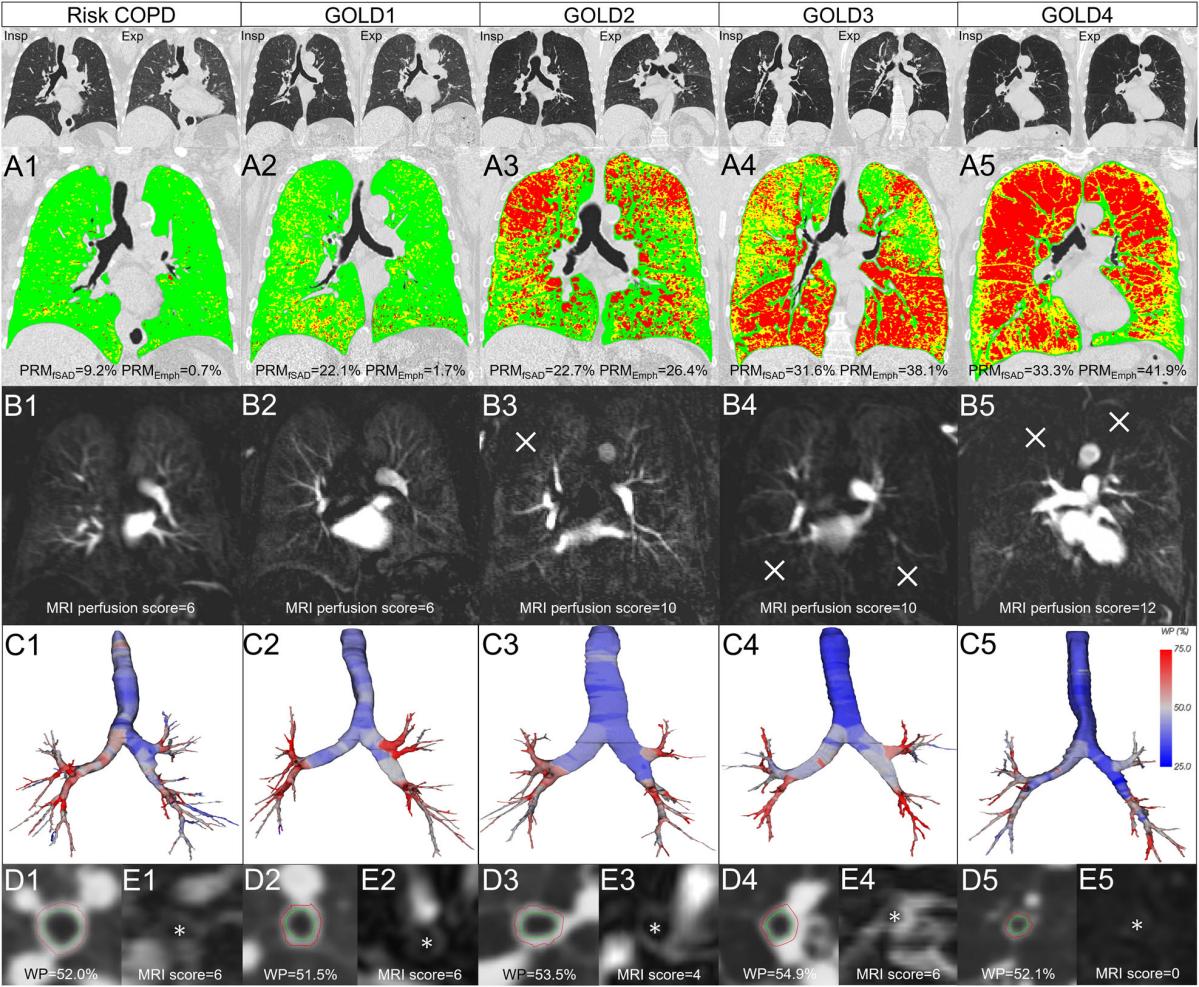
GOLD stage-specific imaging characteristics in QCT and MRI. **A1**–**A5** Quantitative CT images with parametric response imaging (PRM) with normal lung (green = PRM_Normal_), functional SAD (yellow = PRM_fSAD_), and emphysema (red = PRM_Emph_). Note: air trapping and emphysema increase with the GOLD stage. **B1**–**B5** Perfusion MRI images showing homogeneous lung signal in GOLD1 and extensive perfusion defects (white cross) in GOLD4. Note: MRI perfusion defects correspond to CT emphysema. **C1**–**C5** Segmented airway tree on CT (blue = low WP and red = high WP). **D1**–**D5** Axial images of lobe bronchi on CT with segmented airway walls with the highest WP in GOLD3. **E1**–**E5** Transverse T1 VIBE images of lobe bronchi on MRI. Note: assessment of the airway with MRI is much more challenging than with CT

TLV and EI were higher, while the MLD was lower with increasing GOLD grade, indicating increasing emphysema with hyperinflation. Accordingly, PRM_Normal_ showed significantly lower, while PRM_fSAD_, PRM_Emph,_ and PRM_Abnormal_ showed significantly higher values with increasing GOLD grades (all *p* < 0.001). The BE increased from GOLD1 to GOLD4 (*p* = 0.052). In the central airways (generation 1–2), WT_1–2_, TD_1–2_, LA_1–2_, and WP_1–2_ showed no significant differences between the GOLD grades (*p* = 0.498, *p* = 0.673, *p* = 0.530, and *p* = 0.128). In the combined lobar and segmental airways (generations 3–5) and subsegmental airways (generations 6–10), LA_3–5_ was significantly lower in GOLD4, while no other significant differences were observed between GOLD grades. WT and WP tended to be higher from GOLD1 to GOLD3 and lower again in GOLD4, probably indicating airway inflammation and the transition to end-stage airway wall degeneration (Figs. 3 and  4, and Table 2). Further details on the generation-based results can be found in the online supplementary material (Supplemental Table 5).

**Table 2:** Quantitative CT imaging results for the total lung

Quantitative CT	All GOLD grades	Risk COPD	GOLD1	GOLD2	GOLD3	GOLD4	*p*
Parenchyma							
TLV [L]	6.48 (5.40–7.61)	5.22 (4.41–6.72)	6.44 (5.52–7.60)^*^	6.62 (5.55–7.66)	6.56 (5.58–7.64)	7.36 (6.10–8.46)	< 0.001
MLD [HU]	−847 (−864 to 822)	−815 (−832 to 794)	−834 (−851 to 818)^*^	−849 (−862 to 826)^*^	−855 (−870 to 839)^*^	−874 (−888 to 861)^*^	< 0.001
EI [%]	12 (3–25)	1 (0–4)	6 (3−14)^*^	11 (5−21)	20 (11−29)^*^	35 (27–44)^*^	< 0.001
PRM_Normal_ [%]	56 (39–77)	86 (75–93)	72 (62–80)^*^	56 (42–73)^*^	41 (32–56)^*^	27 (22–33)^*^	< 0.001
PRM_fSAD_ [%]	30 (19–38)	12 (6–19)	20 (14–31)	29 (20–38)^*^	36 (29–44)^*^	37 (34–45)	< 0.001
PRM_Emph_ [%]	8 (2–20)	1 (0–2)	3 (2–10)^*^	8 (3–17)^*^	17 (9–25)^*^	31 (22–40)^*^	< 0.001
PRM_Abnormal_ [%]	43 (22–59)	14 (6–23)	26 (18–36)^*^	42 (25–55)^*^	57 (42–66)^*^	71 (66–77)^*^	< 0.001
Airways							
BE	0.60 (0.36–0.96)	0.66 (0.44–1.04)	0.53 (0.33–0.83)	0.56 (0.33–0.96)	0.59 (0.34–0.94)	0.72 (0.57–1.23)	0.052
Central airways (generation 1–2)							
WT_1–2_ [mm]	2.29 (2.14–2.44)	2.30 (2.18–2.44)	2.26 (2.16–2.40)	2.29 (2.14–2.44)	2.31 (2.15–2.47)	2.23 (2.04–2.42)	0.498
TD_1–2_ [mm]	20.71 (19.3–22.1)	20.48 (18.9–22.3)	21.09 (19.5–22.1)	20.70 (19.4–22.2)	20.72 (19.3–22.0)	20.70 (19.2–21.0)	0.673
LA_1–2_ [mm^2^]	212 (178–248)	208 (169–243)	220 (188–248)	211 (180–252)	211 (179–246)	214 (176–248)	0.530
WP_1–2_ [%]	39.50 (36.92–2.15)	39.83 (37.72–2.21)	38.46 (36.67–1.67)	39.57 (36.78–2.08)	40.07 (37.80–2.65)	38.06 (36.21–1.50)	0.128
Large airways (generation 3–5)							
WT_3–5_ [mm]	1.76 (1.6–2.0)	1.81 (1.58–2.00)	1.73 (1.53–1.88)	1.76 (1.59–1.96)	1.76 (1.58–1.96)	1.65 (1.48–1.83)	0.121
TD_3–5_ [mm]	10.28 (9.63–11.02)	10.47 (9.69–11.19)	10.05 (9.70–10.79)	10.44 (9.74–11.06)	10.13 (9.54–10.83)	9.97 (9.05–10.70)	0.027
LA_3–5_ [mm^2^]	40.66 (33.81–8.64)	41.49 (32.78–0.38)	39.90 (34.22–6.69)	42.02 (34.95–0.31)	38.68 (33.22–6.06)	39.85 (31.80–5.83)	0.094
WP_3–5_ [%]	55.95 (51.80–0.12)	55.67 (51.27–0.70)	55.52 (51.47–9.45)	55.7 (51.69–9.71)	57.17 (53.14–0.79)	54.06 (50.34–9.68)	0.217
Subsegmental airways (generation 6–10)							
WT_6–10_ [mm]	0.86 (0.7–1.1)	0.85 (0.68–1.14)	0.83 (0.74–0.95)	0.86 (0.74–1.10)	0.91 (0.74–1.14)	0.87 (0.78–1.00)	0.148
TD_6–10_ [mm]	5.67 (5.25–6.18)	5.72 (5.19–6.33)	5.45 (5.17–5.87)	5.66 (5.29–6.19)	5.75 (5.29–6.23)	5.67 (5.36–6.11)	0.069
LA_6–10_ [mm^2^]	12.66 (11.01–4.83)	13.33 (11.15–4.84)	12.21 (10.54–3.79)	12.67 (11.01–4.72)	12.54 (11.01–5.29)	12.94 (11.11–5.10)	0.322
WP_6–10_ [%]	50.38 (45.41–6.10)	47.66 (43.32–6.81)	49.41 (46.05–3.52)	50.39 (45.43–5.69)	51.23 (46.32–7.31)	50.85 (46.91–5.32)	0.160

Quantitative CT parameters for TLV, EI, MLD, PRM (PRM_Normal_, PRM_fSAD_, PRM_Emph_, and PRM_Abnormal_), BE, WT (WT_1–2_, WT_3–5_, and WT_6–10_), TD (TD_1–2_, TD_3–5_, and TD_6–10_), LA (LA_1–2_, LA _3–5_, and LA _6–10_), and WP (WP_1–2_, WP_3–5_, and WP_6–10_) were calculated. Generation-based results were consolidated into values for the airway central (G_1–2_), large (G_3–5_), and subsegmental (G_6–10_) airway generations. Results are shown for all patients and GOLD grades (risk COPD, GOLD1, GOLD2, GOLD3, and GOLD4). All data are given as median with interquartile range (Q1–Q3)

^*^ *p* < 0.05 vs previous GOLD grade

### GOLD grade-specific semiquantitative MRI scoring

The MRI parenchymal score increased with each GOLD grade from GOLD2 to GOLD4 (*p* < 0.001), while the perfusion defect score was higher overall and increased with each GOLD grade from GOLD1 to GOLD4 (*p* < 0.001). The MRI total central airway disease score and the subscores for tracheal pathologies and main stem pathologies had a median of 0.0 (0.0–0.0) in all GOLD grades. The MRI total large airway disease score and the subscore for large airway disease (bronchiectasis, wall thickening) increased from GOLD1 to 3 and was lower in GOLD4 (*p* < 0.001). The MRI subscore for expiratory airway collapse in large airway and SAD had a median score of 0.0 (0.0–0.0) in all GOLD grades. The MRI airway score including all airway subscores, was lower in risk COPD and GOLD1, higher in GOLD2 and 3, and again lower in GOLD4 (*p* = 0.010). The MRI global score increased with higher GOLD grades (*p* < 0.001) (Figs. 3 and 4, and Table 3).

**Table 3:** Semiquantitative MRI results for the total lung

Semiquantitative MRI	All GOLD grades	Risk COPD	GOLD1	GOLD2	GOLD3	GOLD4	*p*
Parenchymal disease							
Parenchymal defects	0.0 (0.0–2.0)	0.0 (0.0–0.0)	0.0 (0.0–0.0)	0.0 (0.0–2.0)	2.0^*^ (0.0–4.0)	3.0 (0.0–5.0)	< 0.001
Functional disease							
Perfusion defects	9.0 (6.0–11.0)	6.0 (5.3–6.8)	6.0 (6.0–8.0)	9.0^*^ (6.0–11.0)	10.0^*^ (8.0–11.0)	11.0^*^ (10.0–12.0)	< 0.001
Central airways disease							
Trachea	0.0 (0.0–0.0)	0.0 (0.0–0.0)	0.0 (0.0–0.0)	0.0 (0.0–0.0)	0.0 (0.0–0.0)	0.0 (0.0–0.0)	0.694
Main stems	0.0 (0.0–0.0)	0.0 (0.0–0.0)	0.0 (0.0–0.0)	0.0 (0.0–0.0)	0.0 (0.0–0.0)	0.0 (0.0–0.0)	0.151
Total central airway disease	0.0 (0.0–0.0)	0.0 (0.0–0.0)	0.0 (0.0–0.0)	0.0 (0.0–0.0)	0.0 (0.0–0.0)	0.0 (0.0–0.0)	0.191
Large airways disease							
Wall thickening/bronchiectasis	5.0 (2.0–6.0)	4.0 (2.0–6.0)	4.0 (2.0–6.0)	5.0 (2.0–6.0)	6.0 (4.0–6.0)	5.0 (3.0–6.0)	< 0.001
Expiratory airway collapse	0.0 (0.0–0.0)	0.0 (0.0–0.0)	0.0 (0.0–0.0)	0.0 (0.0–0.0)	0.0 (0.0–0.0)	0.0 (0.0–0.0)	> 0.809
Total large airway disease	5.0 (2.0–6.0)	4.0 (2.0–6.0)	4.0 (2.0–6.0)	5.0 (2.0–6.0)	6.0 (3.0–6.0)	4.5 (2.0–6.0)	0.005
SAD							
CLN and PBE	0.0 (0.0–0.0)	0.0 (0.0–0.0)	0.0 (0.0–0.0)	0.0 (0.0–0.0)	0.0 (0.0–0.0)	0.0 (0.0–0.0)	0.011
Scores							
Airway score	6.00 (3.00–6.0)	4.0 (2.00–6.0)	4.0 (2.00–6.0)	6.0 (3.00–6.0)	6.0 (4.00–6.0)	5.0 (3.00–6.0)	0.010
Global score	14.0 (10.0–16.0)	10.0 (6.75–13.0)	10.0 (8.0–13.0)	14.0 (10.25–18.0)	16.0 (12.0–19.0)	19.0 (14.0–22.0)	< 0.001

MRI semiquantitative scores for parenchymal- and perfusion defects, central airway disease (trachea wall thickening/expiratory collapse, main stem wall thickening/expiratory airway collapse), large airway disease (wall thickening/bronchiectasis and expiratory airway collapse) and small airways disease (CLN and PBE), airway score, and the global scores (perfusion defects + airways score) are shown for all GOLD grades (risk COPD, GOLD1, GOLD2, GOLD3, and GOLD4). All data are given as median with interquartile range (Q1–Q3)

*CLN* centrilobular nodules

^*^ *p* < 0.05 vs previous GOLD grade

### GOLD-grade specific characterization of disease heterogeneity

QCT and semiquantitative MRI showed regional differences between upper and LLR in the lung parenchyma and airways. In QCT, PRM_fSAD_ and PRM_Emph_ showed a significant upper lung region predominance in all GOLD grades, except PRM_fSAD_ in GOLD4 (*p* = 0.103), and PRM_Emph_ in GOLD3 and GOLD4 (*p* = 0.176, *p* = 0.474). BE was significantly higher, while WP tended to be higher in the LLR in all GOLD grades. Semiquantitative MRI scoring showed no clear regional predominance of parenchymal and perfusion defects, whereas large airway wall thickening/bronchiectasis was significantly higher in the LLR in all GOLD grades.

### Correlation between semiquantitative MRI and QCT and lung function parameters

**Fig. 5:**
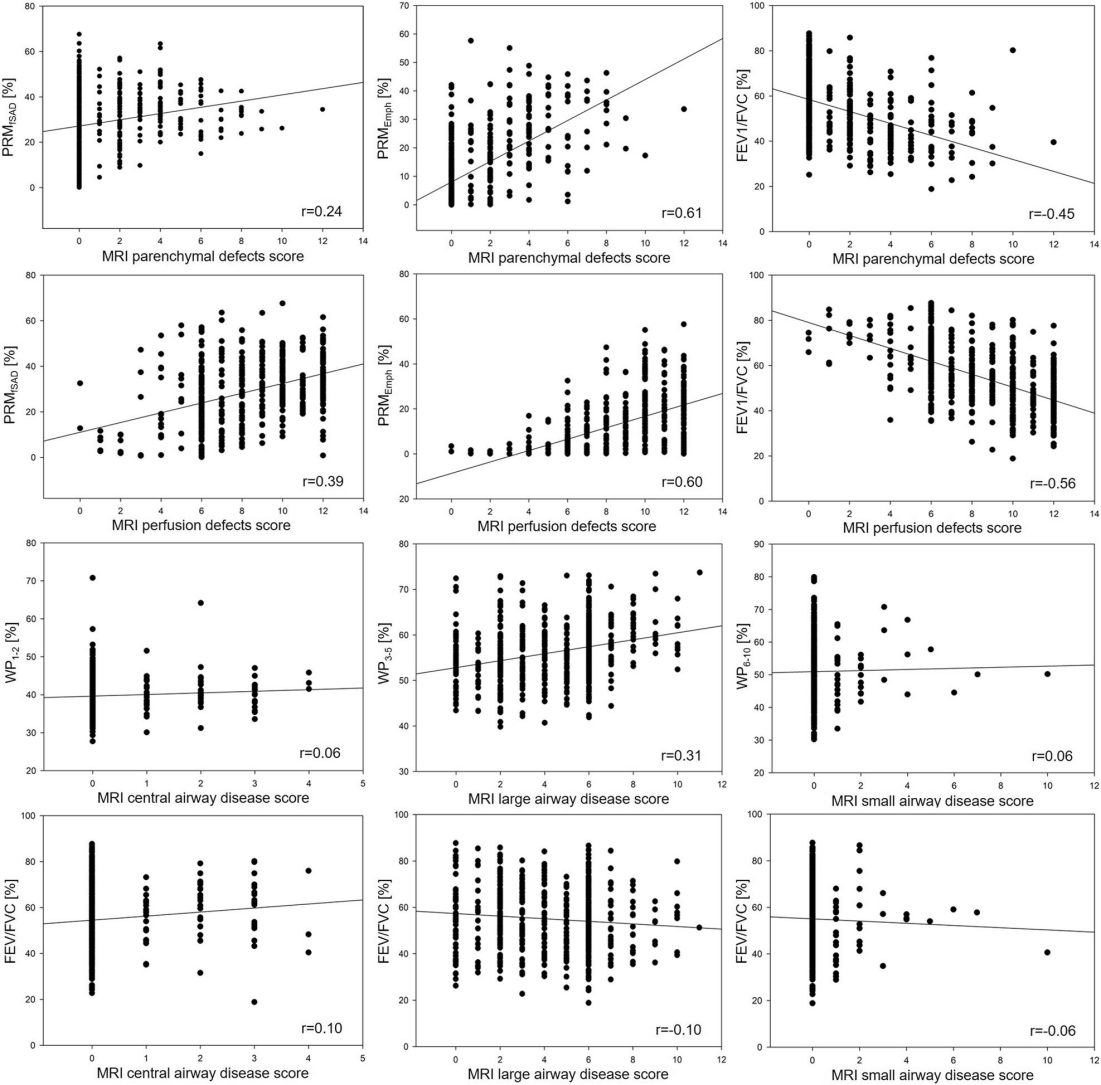
Spearman rank order correlation coefficients. Spearman rank order correlation coefficients were calculated between semiquantitative MRI scores for parenchymal disease (parenchymal and perfusion defect scores), QCT PRM (PRM_fSAD_, PRM_Emph_), and the ratio of FEV_1_/FVC. In addition, semiquantitative MRI scores for central, large, and SAD were correlated with QCT WP pooled for central (WP_1–2_), large (WP_3–5_), subsegmental (WP_6–10_) airways, and FEV_1_/FVC. Spearman’s *r* correlation coefficients are given in each panel. Solid lines indicate the linear regression

The MRI scores for parenchymal and perfusion defects correlated weakly with PRM_fSAD_ (*r* = 0.24, *r* = 0.39), moderately with PRM_Emph_ (*r* = 0.61, *r* = 0.60), and FEV1/FVC (*r* = 0.45, *r* = 0.56). Spearman correlations were calculated for different airway sizes. MRI central airway disease score and QCT WP pooled for central airway generations (WP_1–2_) correlated negligibly (*r* = 0.06), MRI large airway disease score and WP pooled for large airway generations (WP_3–5_) correlated weakly (*r* = 0.31), and MRI SAD score and WP pooled for subsegmental airway generations (WP_6–10_) correlated negligibly (*r* = 0.06). The correlations between MRI airway disease scores and FEV1/FVC were all negligible (*r* = 0.10, *r* = 0.10, and *r* = 0.06) (Fig. 5 and Supplemental Table 6).

## Discussion

The results demonstrate the feasibility of lung MRI for GOLD grade-specific characterization of parenchymal and airway disease in a heterogeneous COPD population. Parenchymal disease scores on MRI were consistent with QCT, as PRM_fSAD_, PRM_Emph_, and MRI parenchymal and perfusion scores were higher in each GOLD grade. MRI subscore for large airway disease indicated airway inflammation in GOLD3 with the transition to airway wall degeneration in GOLD4. Moderate correlations were achieved between MRI parenchymal and perfusion defects vs PRM_Emph_ and FEV1/FVC. MRI large airway disease scores correlated weakly with WP_3–5_ and negligibly with FEV1/FVC.

The quantitative CT parameters TLV, EI, PRM_fSAD_, PRM_Emph_, and PRM_Abnormal_ increased and MLD decreased with higher GOLD grades, which is consistent with the progression of emphysema and hyperinflation [32, 33] (Table 2). Accordingly, the MRI parenchymal and perfusion defect scores were also higher with the increasing GOLD grade, demonstrating that MRI can visualize parenchymal disease progression in COPD (Table 3). However, MRI perfusion defect scores were higher than MRI parenchymal defect scores in all GOLD grades, which can be explained by the different underlying pathophysiological processes representing perfusion and parenchymal defects. Parenchymal defects represent emphysematous destruction and hyperinflation and are reflected by PRM_Emph_ on QCT. Perfusion defects are due to impaired ventilation and hypoxic pulmonary vasoconstriction due to SAD or direct emphysematous destruction of the alveolar capillaries, being a mixture of emphysema and SAD (air-trapping) on QCT (PRM_Emph_ + PRM_fSAD_ = PRM_Abnormal_). Therefore, MRI perfusion defects should affect a larger proportion of the lung than MRI parenchymal defects. QCT results support this observation, as air trapping is the leading pathology in the lower GOLD grades, explaining the significantly higher MRI perfusion defect scores at risk COPD, GOLD1, and GOLD2. Furthermore, technical factors must be considered. Parenchymal defects were recorded as signal voids on contrast-enhanced 3D transversal gradient-echo images, but lung tissue consists mainly of air-filled alveolar space with low hydrogen content and multiple air-tissue surfaces, resulting in low signal intensity and poor signal-to-noise ratio [34]. Therefore, even lower signal intensities in emphysematous areas are difficult to detect visually, suggesting that a dynamic MRI sequence for perfusion defect detection may be necessary to achieve satisfactory sensitivity for subtle emphysema and air trapping, especially in lower GOLD grades. Another finding was a faster increase in MRI parenchymal and perfusion defects between higher GOLD grades, suggesting an accelerated progression of emphysema in advanced COPD, possibly due to the effect that the locally highly altered alveolar micromechanics within a damaged lung could itself become an “independent trigger for the progression of lung damage [35].

QCT airway parameters showed no significant differences between GOLD grades, but WP for central, large, and small airways tended to be higher in GOLD2 and GOLD3 and lower in GOLD4, which is consistent with other work from our group, including a comparable subpopulation from the COSYCONET trial [36–38] (Table 2). However, the literature does not yet provide a consistent picture of the chronological development of airway disease in the different GOLD grades. SAD is considered a central feature of COPD, with chronic inflammation leading to progressive narrowing and destruction of the small airways [39–41]. Koo et al demonstrated a significant loss of terminal and transitional bronchioles in GOLD1 and GOLD2, as the remaining small airways showed thickened walls and narrowed lumens which were also present in regions without emphysema [42]; other studies have found significantly reduced airway WT in most areas of the central tracheobronchial tree in COPD patients, with possible mechanisms including regression of airway smooth muscle, apoptosis or replacement fibrosis, and reduced bronchial vascular volume [43–45]. Therefore, we interpreted the changes in airway dimensions observed as a possible transition from reversible airway inflammation in GOLD2 to GOLD3 to irreversible airway damage with degradation of the airway walls in GOLD4. This study compared semiquantitative MRI and quantitative CT for airway disease in different GOLD grades. We tried to achieve better comparability by combining the QCT airway parameters for central (generation 1–2), large = lobar and segmental (generation 3–5), and subsegmental (generation 6–10) airways, as the MRI scoring system used an analogous categorization by distinguishing between central, large and SAD. The medians of the MRI subscores for central and SAD were zero for all GOLD grades, so a comparison with the corresponding QCT parameters was not possible (Table 3). At least, the MRI subscore for large airway disease showed a comparable trend to the WP_3–5_, with the highest scores in GOLD3, and again lower scores in GOLD4, possibly reflecting also airway inflammation and the transition to airway degeneration. Our results showed the limitations of MRI in the assessment of airway pathologies, in particular, central and peripheral airways were difficult to assess, mainly due to the higher vulnerability to respiratory and cardiac motion and the lower spatial resolution of MRI [20, 46]. Therefore, the best results are obtained for large airways (lobar and segmental bronchi), as they are better visualized on MRI images.

In conclusion, semiquantitative lung MRI can provide GOLD grade-specific characterization of parenchymal and airway disease. Parenchymal disease increased with higher GOLD grades as air trapping and emphysema increased. MRI perfusion defect scores were higher than MRI parenchymal defect scores in all GOLD grades, as perfusion defects represent a mixture of air trapping and emphysema. Furthermore, air trapping is the leading parenchymal pathology in lower GOLD grades, which explains the significantly higher MRI perfusion defect scores at risk COPD, GOLD1, and GOLD2. MRI assessment of airway pathology was limited for the central and peripheral airways due to susceptibility to respiratory and cardiac motion and low spatial resolution. However, MRI large airway disease had the highest scores in GOLD3 and again lower scores in GOLD4, possibly reflecting airway inflammation and the transition to airway degeneration.

COPD is a heterogeneous disease characterized by varying regional distribution of parenchymal and airway pathologies. Our regional analysis compared upper and LLR, showing an upper lobe predominance for PRM_fSAD_ and PRM_Emph_ in all GOLD grades, which is consistent with the literature [47]. Accordingly, MRI parenchymal defects and MRI perfusion defects tended to be higher in the upper lobes, but the differences were less pronounced (Table 4). Overall, regional differences in airway involvement are less well studied. In smokers, Tho et al showed significantly lower Pi10 values in the right upper lobe compared to the lower lobes [48]. We also found greater airway involvement in the LLR as the QCT parameter BE was significantly higher and WP tended to be higher in the lower lobes in almost all GOLD grades. Similarly, the MRI score for large airway wall thickening/bronchiectasis showed a significant predominance in the lower lobes in all GOLD grades (Table 4). In conclusion, semiquantitative MRI seems to capture the regional heterogeneity of COPD. The ability to detect regional heterogeneity, i.e., upper or lower lobe predominance, is clinically relevant as it is associated with disease progression and has clinical implications for disease management [49–51]. The more uniform distribution of MRI perfusion defects may be due to it being a precursor to structural changes detectable with CT and is consistent with the literature, as is the spatial distribution of ventilation defects [52]. Gravitational effects due to the supine position of the patient in the scanner were not evaluated, as this is not possible with the lobe-based MRI scoring system used [53]. It is known that the supine position introduces a ventral to dorsal lung attenuation gradient and influences the perfusion blood flow strength [54, 55]. As both the CT and MRI scans were performed in the supine position, this should play a minor role in the comparison between the MRI and CT methods.

**Table 4:** QCT and semiquantitative MRI for the combined upper (ULR) and lower (LLR) lung regions

	All GOLD grades	Risk COPD	GOLD1	GOLD2	GOLD3	GOLD4	*p*
Parenchymal disease							
QCT PRM_fSAD_, [%]							
ULR	34 (24–44)	16 (10–27)	27 (19–39)	35 (26–43)	40 (32–48)	41 (35–47)	< 0.001
LLR	25 (11–36)	5 (2–14)	11 (6–19)	24 (13–35)	32 (25–42)	37 (31–43)	< 0.001
*p*	< 0.001	< 0.001	< 0.001	< 0.001	< 0.001	0.103	
QCT PRM_Emph_, [%]							
ULR	10 (3–20)	1 (0–4)	5 (2–11)	10 (3–19)	17 (9–25)	31 (23–41)	< 0.001
LLR	5 (1–18)	0 (0–1)	2 (1–6)	6 (2–14)	15 (4–25)	28 (20–35)	< 0.001
*p*	< 0.001	< 0.001	< 0.001	< 0.001	0.176	0.474	
QCT PRM_Abnormal_, [%]							
ULR	50 (29–64)	18 (11–30)	33 (25–46)	50 (31–61)	59 (49–69)	75 (65–81)	< 0.001
LLR	33 (12–54)	5 (2–17)	14 (7–25)	33 (15–49)	52 (34–64)	69 (62–74)	< 0.001
*p*	< 0.001	< 0.001	< 0.001	< 0.001	0.176	0.046	
MRI parenchymal defect score							
ULR	0.0 (0.0–0.5)	0.0 (0.0–0.0)	0.0 (0.0–0.0)	0.0 (0.0–0.3)	0.3 (0.0–0.5)	0.5 (0.0–1.0)	< 0.001
LLR	0.0 (0.0–0.0)	0.0 (0.0–0.0)	0.0 (0.0–0.0)	0.0 (0.0–0.0)	0.0 (0.0–0.5)	0.5 (0.0–1.0)	< 0.001
*p*	0.645	0.307	0.052	0.015	0.015	0.644	
MRI perfusion defect score							
ULR	1.5 (1.0–2.0)	1.0 (1.0–1.0)	1.0 (1.0–1.5)	1.5 (1.0–2.0)	1.5 (1.3–2.0)	2.0 (1.5–2.0)	< 0.001
LLR	1.5 (1.0–2.0)	1.0 (1.0–1.0)	1.0 (1.0–1.0)	1.5 (1.0–2.0)	2.0 (1.0–2.0)	2.0 (2.0–2.0)	< 0.001
*p*	< 0.001	0.168	0.300	0.432	0.145	0.274	
Airway disease							
QCT BE							
ULR	0.24 (0.11–0.49)	0.28 (0.15–0.64)	0.18 (0.10–0.32)	0.26 (0.13–0.55)	0.21 (0.10–0.44)	0.22 (0.09–0.61)	0.018
LLR	1.19 (0.75–1.90)	1.29 (0.85–1.98)	1.02 (0.73–1.48)	1.18 (0.69–1.83)	1.28 (0.75–2.00)	1.32 (0.86–2.36)	0.159
*p*	< 0.001	< 0.001	< 0.001	< 0.001	< 0.001	< 0.001	
QCT WP, [%]							
ULR	53.69 (48.5–58.7)	52.57 (47.3–59.5)	52.21 (46.6–57.8)	53.61 (48.2–58.0)	54.99 (49.7–60.3)	53.99 (49.4–60.3)	0.098
LLR	54.79 (49.4–60.5)	51.65 (47.7–60.7)	54.67 (49.9–59.1)	54.79 (49.8–59.7)	55.54 (50.6–61.7)	54.32 (48.8–58.3)	0.104
*p*	0.007	0.940	0.110	0.008	0.157	0.777	
MRI large airway disease score							
ULR	0.8 (0.3–1.0)	0.5 (0.0–1.0)	0.5 (0.0–1.0)	0.8 (0.3–1.0)	1.0 (0.5–1.0)	0.8 (0.4–1.0)	< 0.001
LLR	1.0 (1.0–1.0)	1.0 (1.0–1.0)	1.0 (1.0–1.0)	1.0 (1.0–1.0)	1.0 (1.0–1.0)	1.0 (1.0–1.0)	0.090
*p*	< 0.001	< 0.001	< 0.001	< 0.001	< 0.001	0.005	

Quantitative CT parameters for PRM (PRM_Normal_, PRM_fSAD_, PRM_Emph_, PRM_Abnormal_), the airway parameters BE and WP (WP_3–10_), and the MRI semiquantitative scores for parenchymal and perfusion defects and large airway disease (wall thickening/bronchiectasis) are shown for the upper and LLR in different GOLD grades (risk COPD, GOLD1, GOLD2, GOLD3, and GOLD4). All data are given as median with interquartile range (Q1–Q3)

^*^ *p* < 0.05 vs previous GOLD grade

Correlations between MRI scores and QCT were higher for MRI parenchymal and perfusion defects scores than for MRI airway disease subscores. The MRI scores for parenchymal and perfusion defects correlated weakly with PRM_fSAD_, but moderately with PRM_Emph_ (*r* = 0.61) (Fig. 5). We believe these results are satisfying as we have shown only slightly higher correlations (*r* = 0.75) between PRM_Emph_ and MRI perfusion defects in percent (QDP), the latter calculated by using a computer-assisted quantification method in a single-center substudy [56]. The correlations between PRM_fSAD_ and MRI perfusion defects were weak, suggesting that visual detection of air trapping on MRI may be difficult and lead to underscoring, mainly at lower GOLD grades. We correlated QCT and MRI airway disease by pooling the QCT data for WP for central (generations 1–2), large (generations 3–5), and subsegmental airways (generations 6–10) and matching them with the corresponding MRI subscores. As expected, the correlations between MRI scores for central airway disease and WP_1–2_ and between MRI SAD and WP_6–10_ were negligible due to respiratory and cardiac motion and low spatial resolution. Only MRI large airway disease score and (WP_3–5_) showed a weak correlation (Fig. 5). It seems possible that the weak correlation is partly due to the MRI scoring system, as most airway pathologies were scored on a 3-point scale or for presence or absence on less than or more than 50% of a lung lobe, which provided a description of presence rather than detailed quantitative information, which probably resulted in lower coefficients when correlated with numerical QCT values.

Lung function parameters showed the expected impairment associated with GOLD grades, with TLC and RV significantly higher in GOLD4 than in GOLD1, and FVCpp and FEV1 percent predicted (FEV1pp) lower from GOLD1 to GOLD4. However, FVCpp and FEV1pp were lower in “risk COPD” than in GOLD1. The same observation was made by Karch et al who analyzed the total COSYCONET study population (*n* = 2741) [21]. They concluded that the administration of high-dose bronchodilators (used to standardize the patient’s condition prior to the functional assessments) may have improved FEV1/FVC above the thresholds of ≥ 0.7 in some GOLD1 patients, leading to their classification as “risk COPD”, possibly influencing the median FEV1pp in the latter group. MRI scores for parenchymal and perfusion defects correlated moderately with FEV1/FVC (*r* = −0.45 and *r* = −0.56), which is slightly lower than the correlation between PRM_Emph_ and FEV1/FVC (*r* = −0.72) in a comparable study [37]. However, the results indicate a relationship between parenchymal disease and airflow limitation. The best correlations (moderate) were obtained between perfusion defects and FEV1/FVC, as a reduction in FEV1/FVC is mainly due to small airway obstruction, and perfusion defects represent emphysema and SAD [57, 58]. The correlation between FEV1%pred and MRI parenchymal and perfusion defect was also moderate [59]. The correlations between MRI airway disease scores and FEV1/FVC were all negligible (*r* = 0.10, *r* = 0.10, and *r* = 0.06) (Fig. 5 and Supplemental Table 6).

The study had the following limitations. (1) Parenchymal and perfusion defects on MRI may be influenced by lung mass, pulmonary embolism, pneumonia, and other comorbidities such as interstitial lung disease or cystic fibrosis. Major exclusion criteria for the imaging substudy were previous lung surgery (e.g., lung volume reduction or lung transplantation) and moderate or severe exacerbations requiring antibiotic treatment within the previous four weeks. In addition, interstitial lung disease, fibrosis, or cystic fibrosis were not reported as comorbidities in the COSYCONET baseline report, suggesting at least a low prevalence in the imaging subcohort [21]. Pulmonary masses (> 3 cm), central or peripheral pulmonary emboli, or other opacities were not part of the MRI scoring system but were not observed by visual assessment of the MRI images. However, pulmonary nodules were a common finding. A single pulmonary nodule was reported in 227 patients (mean size 7 mm), two pulmonary nodules were reported in 105 patients (mean size 6 mm), and three pulmonary nodules were reported in 52 patients (mean size 5 mm) [60]. Overall, we believe that the pulmonary nodules had little effect on the parenchymal and perfusion defect scores due to their small volume. (2) MRI airway disease scores for central and small airways showed zero medians for all GOLD grades. This was unsatisfying as it was not possible to compare the results with the QCT parameters. In addition to the fact that central and small airways are more difficult to score visually on MRI due to technical limitations, the sample size may have been underpowered. COSYCONET was originally powered at 90% to detect risk factors (especially comorbidities) that increased the odds ratio of relevant BODE worsening by more than 1.5. The final sample size (*n* = 2741 at visit 1, *n* = 2000–2200 expected at visit 3) was smaller than the originally planned sample size (*n* = 3000 at visit 3), but still provides 70–80% power to detect odds ratios greater than 1.5 [21]. In this context, it was not possible to calculate the power required for our imaging substudy, as the effect sizes of the MRI findings were unknown. In addition, the number of patients in the imaging substudy (*n* = 625) was set, as it was the maximum within the available resources. However, it is possible that the MRI findings of the central and small airways would have been more informative with a larger sample size. (3) The MRI score as presented has limitations, particularly in some subscores that seem difficult to objectify, resulting in poor inter-reader agreement (Supplemental Table 4). In this context, it would have been useful to perform an intra-reader reproducibility study, but this was not possible due to the high personnel requirements. However, the results could have led to more targeted training of readers, which would ultimately have reduced inter-reader variability. We attempted to overcome this drawback by using two first readers who were reviewed by a third reader with more than 20 years of experience in lung MRI as an adjudicator to reach a consensus. In the future, adding computer-assisted quantitative parameters for MRI could improve the consistency and objectivity of the scores. However, a reliable quantitative MRI assessment is under development, but not yet available for the multicenter COSYCONET study, mainly because of the high requirements on a potential software with regard to variations in data quality. (4) QCT and MRI imaging in large networks or cohort studies often face the problem of heterogeneous equipment. In most cases, the true extent and underlying causes of inter-center imaging biomarker variability can only be estimated. In the present multi-center study, all current recommendations were followed to ensure the highest possible degree of standardization. However, despite all efforts, a possible imaging center-induced bias cannot be completely excluded.

In conclusion, semiquantitative lung MRI can provide GOLD grade-specific characterization of parenchymal and airway disease, although the sensitivity of MRI in detecting subtle parenchymal changes and airway disease is lower than that of QCT. The correlation between MRI scores and QCT and lung function parameters was moderate for MRI parenchymal disease and weak for MIR airway disease, limiting the usefulness of MRI in assessing airway disease. In addition, the semiquantitative scoring system may lead to subjectivity and consistency issues, as some MRI scores showed poor inter-reader agreement, which may affect the reliability of the results. In the future, the addition of computer-assisted quantitative parameters for MRI may improve the consistency and objectivity of MRI imaging features. Therefore, MRI can be used as a radiation-free imaging modality in scientific and clinical settings, provided that its capabilities and limitations are carefully considered.

## Supplementary information

ELECTRONIC SUPPLEMENTARY MATERIAL

## Abbreviations

BE: Bronchiectasis index
COPD: Chronic obstructive pulmonary disease
EI: Emphysema index
FEV1: Forced expiratory pressure in 1 s
FEV1/FVC: Ratio of FEV1 to FVC in percent
FEV1pp: FEV1 percent predicted
FVC: Forced vital capacity
GOLD: Global initiative for obstructive lung disease
HU: Hounsfield units
LA: Lumen area
LLi: Lingula
LLL: Left lower lobe
LLR: Lower lung regions
LUL: Left upper lobe
MLD: Mean lung density
PBE: Peripheral bronchiectasis
PFT: Pulmonary function test
PRM: Parametric response mapping
QCT: Quantitative computed tomography
RLL: Right lower lobe
RML: Middle lobe
RUL: Right upper lobe
RV: Residual volume
SAD: Small airway disease
TD: Total diameter
TLC: Total lung capacity
TLV: Total lung volume
ULR: Upper lung regions
WP: Wall percentage
WT: Wall thickness
