# Supplementary material for: GOLD grade-specific characterization of COPD in the COSYCONET multi-center trial: comparison of semiquantitative MRI and quantitative CT
Source: Eur Radiol. 2025 Jan 8;35(7):3842–57. doi: 10.1007/s00330-024-11269-3 (PMC12165896; doi:10.1007/s00330-024-11269-3)
Supplement: Supplementary file 1 — ELECTRONIC SUPPLEMENTARY MATERIAL [file 330_2024_11269_MOESM1_ESM.pdf]

GOLD grade-specific characterization of COPD in the COSYCONET Multi-center Trial: Comparison of semiquantitative MRI and quantitative CT

ELECTRONIC SUPPLEMENTARY MATERIAL

CT image acquisition

CT were acquired by clinical CT scanners from three different manufacturers with at least 40-line detector arrays (Siemens Healthineers, General Electric, Philips). The standardized, non-enhanced, low-dose CT protocol included full inspiratory and end-expiratory spiral acquisitions at a thin-slice collimation of 0.6 mm, a pitch of 0.6 - 1.0, a tube potential of 120 kVp, and current of 35 effective mAs with only minor adaptions to scanner type (Supplemental Table 1). CT images were reconstructed with smooth and edge-enhancing algorithms (B70f/LUNG/L and B30f/SOFT/B, generic names of Siemens/General Electric/Philips). The maximum effective dose of paired low-dose CT was less than 3.5 mSv. Continuous assessment of image quality throughout the study was monitored using a commercial CT phantom (Catphan600, The Phantom Laboratory).

MRI image acquisition

MRI examinations were performed on clinical MR systems with 1.5 T (Magnetom Aera, Avanto, Espree and Symphony, Siemens Healthineers) or 3.0 T (Magnetom Trio, Siemens Healthineers and Ingenia, (Philips). A general MRI protocol for the assessment of structural and functional lung changes in COPD patients was composed as suggested in previous work and adapted to the specifications of each scanner as necessary [1; 2]. The total image acquisition time was approximately 30 min. MRI included morphological non-enhanced and contrast-enhanced sequences in in- and expiration and a DCE-MRI sequence to study lung perfusion. The protocol was designed to be applicable at all scanners in the different locations of the study. Therefore, it was based on commercially available sequences like 3D gradient echo (GE) and fast spin echo sequences for morphological imaging. More recent

developments such as ultra-short echo-time sequences were not yet included. The DCE-MRI perfusion imaging used a fixed dose of 2 ml gadolinium-based contrast agent (Gadobutrol, Bayer Vital GmbH), which was injected i.v. at 4 ml/s followed by a saline chaser [3; 4]. The DCE-MRI was acquired in inspiratory breath-hold (Supplemental Table 2). A dedicated phantom was used to document image quality [5].

Supplemental Table 1. Acquisition parameters and image reconstruction protocol for paired inspiratory and expiratory CT.

**Acquisition**	
Scanner models*	GE Lightspeed VCT64/GEOptima64<br>Siemens Definition AS40/64/Flash128<br>Philips Brilliance 64/iCT256
Scan Type	Spiral
Rotation Time [s]	0.33 - 0.50
Collimation [mm]	40 / 64 / 128 x 0.6 - 0.625
Pitch	0.6 - 1.0
kVp	120
mAs	30 - 35
Dose modulation	Off
Matrix	512 x 512
Calibration phantom	Air / water phantom / CatPhan
Max. eff. dose/scan [mSv]	< 1.75
Max. eff. overall dose [mSv]	< 3.50

**Reconstructions**	**Orientation**	**kernel**	**FOV**	**Slice thickness (mm)**	**Interval (mm)**
Inspiratory	axial	sharp / soft	lung	1.25-1.50	0.70-0.75
Inspiratory	axial	sharp / soft	lung	0.625-1.00	0.50
Inspiratory	axial	soft	including soft tissue of torso	0.625-1.00	0.50
Expiratory	axial	sharp / soft	lung	0.625-1.00	0.50

Acquisition parameters and image reconstruction protocol for paired inspiratory and expiratory CT. *Vendor-specific generic names for Siemens/GE/Philips. Note- The protocol was designed based on the Siemens platform, and then adapted to regionally available scanner hardware.

Supplemental Table 2. MRI protocol, designed for 1.5T Siemens MAGNETOM Aera.

Sequences	mode	orientation	breath mode	contrast agent	TR (ms)	TE (ms)	FoV (mm^2)	Slice thick. (mm)	voxel size (mm^2)	matrix	PAT factor	scan time (min:s)
VIBE	3D	cor	insp		3.61	1.63	400×400	4.0	1.39×1.39	288×288	2	0:16
VIBE	3D	tra	insp		3.29	1.61	400×300	4.0	1.25×1.25	320×240	2	0:16
HASTE	2D	cor	insp		314.0	20.0	400×400	6.0	0.78×0.78	512×512	3	0:13
HASTE	2D	tra	2×insp		500.0	27.0	450×366	8.0	1.41×1.41	320×260	2	0:35
HASTE	2D	cor	exp	native	314.0	20.0	400×400	6.0	0.78×0.78	512×512	3	0:13
TrueFISP	2D	cor	free		448.9	1.17	400×400	4.5*	0.78×0.78	512×512	3	2:20
BLADE	2D	cor	5×insp		905.0	73.0	400×400	6.0	1.25×1.25	320×320	2	2:13
HASTE IRM	2D	tra	2×insp		502.0	72.0	400×400	6.0	1.56×1.56	256×256	2	0:38
FLASH	3D	cor	insp		2.80	1.04	350×400	1.8	1.04×1.04	336×384	3	0:16
TWIST	3D	cor	Insp	dynamic	1.73	0.76	366×450	5.0	1.76×1.76	208×256	2	0:37
FLASH	3D	cor	insp	angio	2.80	1.04	350×400	1.8	1.04×1.04	336×384	3	0:16
VIBE FatSat	3D	tra	Insp		3.29	1.61	400×300	4.0	1.25×1.25	320×240	2	0:17
VIBE FatSat	3D	tra	exp	post-	3.29	1.61	400×300	4.0	1.25×1.25	320×240	2	0:17
VIBE	3D	cor	Insp	contrast	3.61	1.63	400×400	4.0	1.39×1.39	288×288	2	0:16
VIBE	3D	cor	exp		3.61	1.63	400×400	4.0	1.39×1.39	288×288	2	0.16

The parameters shown are acquisition mode (3D or 2D multi-slice), slice/slab orientation, breathing mode (inspiration, expiration or free breathing and number of breath-holds), status of contrast agent during acquisition, repetition time TR, echo time TE, Field of View, slice thickness, in-plane resolution/voxel size, matrix size, parallelization factor and total scan time. *The slices in the balanced SSFP (TrueFISP) sequence were acquired with 60% overlap.

Quantitative post-processing of CT images

A validated scientific software package (YACTA v2.8.2.3) was used for fully automated segmentation of the airway tree and lung lobes on both, inspiratory and expiratory [6-9]. Emphysema was quantified by total lung volume (TLV), mean lung density (MLD) and the emphysema index (EI), based on the accepted threshold of -950 HU [10; 11]. TLV, MLD and EI increase with emphysema progression. Parametric response mapping (PRM) was performed after deformable CT volume registration, which allows the combination of inspiratory and expiratory CT lung scans to classify individual lung parenchyma voxels as normal (PRM_{Normal}), voxels with functional small airways disease (PRM_{fSAD}) and emphysema (PRM_{Emph}) by assuming that lung voxels with inspiratory CT attenuation less than -950 HU represent emphysema, while voxels with values greater than -950 HU on inspiration but less than -856 HU on expiration represent functional small airway disease (fSAD) [12; 13]. $PRM_{Abnormal}$ was calculated $PRM_{Abnormal} = PRM_{fSAD} + PRM_{Emph}$. All variables were computed for the total lung and all lobes separately (i.e. right upper (RUL), middle (RML) and lower (RLL) lobe, as well as left upper lobe (LUL), lingula (LLi) and left lower lobe (LLL) and for the combined upper (ULR = RUL + RML + LUL + LLi) and lower (LLR = RLL + LLL) lung regions. The airways were analyzed using an airway generation- and lobe-based approach. Airway generations were the trachea (G1), right and left main stem (G2), lobar (G3), segmental (G4 and G5), and the individual subsegmental bronchi (G6 to G10). Airway results were simplified by consolidating the generation-based results for the combined central airways (G_{1-2}), large (lobar- and segmental airways) (G_{3-5}), the subsegmental airways (G_{6-10}). The following measurements were obtained 1) Wall thickness (WT), distance between the inner and outer edges, in mm 2) Total airway diameter (TD), distance between the outer borders of the bronchus, in mm 3) Lumen area (LA), area between the inner borders, in mm^2 4) Wall percentage (WP), ratio of wall thickness to overall diameter, in %. The lobes-based approach calculated the BE and WP in all lung lobes bronchi. Analogue to the analysis of emphysema, the results for the combined upper and lower lung region were pooled.

Semiquantitative MRI assessment

MR images were visually evaluated using OsiriX software (OsiriX 64-bit, Pixmeo SARL) on a dedicated workstation (iMac 27, Apple Inc.) with two 21” certified medical image monitors (Eizo, Nanao Corporation). Two radiologists with 3 years of experience in lung imaging analysed the images independently. Both studies from each patient were read separately by each reader, who was blinded to the images and results from the other modality. A minimum of 2 weeks was allowed between readings of MRI and CT to minimise recall bias. Finally, the records of the two first readers were reviewed by a third reader with more than 20 years of experience in pulmonary MRI as an adjudicator to reach a consensus.

Semiquantitative visual scoring of COPD-related pathologies was performed, using a previously established MRI scoring system in cystic fibrosis and COPD [14; 15]. Lung parenchymal defects and lung perfusion defects were scored on a 3-point scale for all 6 lobes (0=absent, 1=≤50%, 2=>50% of the lobe affected; 0-2 points per lobe = 0-12 point for the total lung). Lung parenchymal defects were recorded as an indicator of pulmonary emphysema, while the affected parts of the lung appeared as signal void on the contrast-enhanced transverse 3D gradient echo image, as perfusion deficits on the DCE series and as a signal void on the transverse half Fourier fast spin echo image. Perfusion deficits representing functional disease (small airway disease and emphysema), appearing as perfusion defects on the DCE series and signal void on the contrast-enhanced 3D transversal gradient echo image during expiration. (Supplementary Table 3). Multiple MRI features of central, large and small airways disease were rated binary (0=not present, 1=present) or using a 3-point-scale for each lobe (0=absent, 1=≤50%, 2=>50% of the airways affected). Central airway disease (wall thickening/expiratory collapse) was scored binary in the trachea and right and left main bronchi. Large airway disease (bronchiectasis/wall thickening) was scored on a 3-point scale per lobe and expiratory collapse of the lobar bronchi was scored binary. Small airway disease (tree-in-bud appearance and peripheral bronchiectasis) was scored on a 3-point scale per lobe (Supplementary Table 3). MRI, a single sum score was given for bronchiectasis and/or bronchial wall thickening, since reporting these separately was expected to be difficult.

Furthermore, bronchial wall thickening appears less conspicuous with a slightly better visualization on the contrast-enhanced transverse 3D gradient echo image.

Supplemental Table 3. Standardized semiquantitative visual evaluation of parenchymal, functional and airway disease on MRI images.

l	MRI features	MRI score	
**Parenchymal disease**	Lung parenchyma defects = Emphysema	0-2 points per lobe (0=absent, 1=≤50, 2=>50% of the lobe affected)	0-12 point for the total lung (6x lobes)
**Functional disease**	Lung perfusion defects = Emphysema + small airway disease	0-2 points per lobe (0=absent, 1=≤50, 2=>50% of the lobe affected)	0-12 point for the total lung (6x lobes)
**Central airway disease**	Wall thickening/ Expiratory collapse trachea	0-1 (0=absent, 1=present)	0-3 points for the total lung
	Wall thickening/ Expiratory collapse right main bronchus	0-1 (0=absent, 1=present)	
	Wall thickening/ Expiratory collapse left main bronchus	0-1 (0=absent, 1=present)	
**Large airway disease**	Wall thickening/bronchiectasis	0-2 points per lobe (0=absent, 1=≤50, 2=>50% of the airways affected)	0-12 point for the total lung
	Expiratory collapse of lobar bronchi	0-1 (0=absent, 1=present)	0-6 point for the total lung
**Small airway disease**	Tree-in-bud appearance/peripheral bronchiectasis	0-2 points per lobe (0=absent, 1=≤50, 2=>50% of the airways affected)	0-12 point for the total lung
	**Airway score**	Central + large + small airway score	0-33 points
	**Global score**	Functional disease + Airway score	0-12 + 0-33 = 0-45 points

Supplemental Table 4. Fleiss kappa for semiquantitative visual evaluation of parenchymal and airway disease.

MRI feature	Fleiss κ (lwr.ci - upr. ci)						
Parenchymal disease	RUL	RML	RLL	LUL	LLi	LLL	Lung
Parenchymal defects	0.22 (0.18-0.26)	0.19 (0.15-0.24)	0.23 (0.19-0.27)	0.25 (0.20-0.29)	0.14 (0.10-0.18)	0.22 (0.18-0.27)	0.14 (0.11-0.17)
Perfusion defects	0.59 (0.54-0.63)	0.58 (0.53-0.63)	0.48 (0.43-0.52)	0.57 (0.52-0.61)	0.59 (0.55-0.64)	0.46 (0.41-0.51)	0.39 (0.37-0.41)

Central airway disease	Trachea	Right main bronchi	Left main bronchi
Wall thickening/ bronchiectasis	0.54 (0.49-0.58)	0.00 (0.05-0.05)	0.00 (0.05-0.05)
Expiratory collapse	0.59 (0.54-0.64)	0.56 (0.51-0.61)	0.60 (0.55-0.64)

Large airway disease	RUL	RML	RLL	LUL	LLi	LLL	Lung
Wall thickening/ bronchiectasis	0.37 (0.32-0.41)	0.33 (0.29-0.38)	0.24 (0.20-0.28)	0.38 (0.33-0.43)	0.37 (0.32-0.41)	0.23 (0.19-0.27)	0.19 (0.16-0.21)
Expiratory collapse	0.00 (0.05-0.05)	0.14 (0.09-0.19)	0.17 (0.12-0.21)	0.00 (0.05-0.05)	0.00 (0.05-0.05)	0.17 (0.13-0.22)	0.17 (0.13-0.20)

Small airway disease	RUL	RML	RLL	LUL	LLi	LLL	Lung
Tree-in-bud appearance/ peripheral bronchiectasis	0.45 (0.40-0.49)	0.38 (0.34-0.43)	0.43 (0.39-0.48)	0.44 (0.40-0.49)	0.43 (0.39-0.48)	0.51 (0.47-0.55)	0.35 (0.32-0.39)

SUPPLEMENTAL RESULTS

Supplemental Table 5. Generation-based QCT airway parameters.

	All GOLD grades	Risk COPD	GOLD1	GOLD2	GOLD3	GOLD4	p
**Airway wall thickness [mm]**							
WT_1	2.39±0.27	2.39±0.25	2.40±0.26	2.38±0.27	2.42±0.28	2.32±0.28	0.252
WT_2	2.18±0.28	2.18±0.28	2.16±0.24	2.18±0.28	2.20±0.29	2.13±0.32	0.707
WT_3	2.13±0.33	2.14±0.35	2.13±0.25	2.12±0.30	2.16±0.36	2.04±0.36	0.291
WT_4	1.83±0.36	1.85±0.36	1.76±0.30	1.84±0.34	1.84±0.38	1.75±0.40	0.240
WT_5	1.36±0.34	1.39±0.37	1.28±0.22	1.36±0.33	1.39±0.38	1.29±0.33	0.147
WT_6	1.07±0.32	1.06±0.31	1.03±0.24	1.07±0.35	1.11±0.32	1.04±0.32	0.393
WT_7	0.93±0.31	0.93±0.30	0.88±0.27	0.92±0.30	0.96±0.33	0.92±0.28	0.478
WT_8	0.82±0.29	0.82±0.32	0.77±0.23	0.80±0.29	0.84±0.30	0.85±0.26	0.553
WT_9	0.77±0.33	0.71±0.31	0.71±0.24	0.79±0.33	0.79±0.31	0.87±0.42	0.084
WT_{10}	0.75±0.39	0.74±0.47	0.65±0.22	0.74±0.38	0.77±0.35	0.83±0.44	0.603
**Airway total diameter [mm]**							
TD_1	23.61±2.48	23.35±2.42	24.06±2.34*	23.57±2.65	23.69±2.33	23.53±2.26	**0.001**
TD_2	17.83±1.97	17.75±2.11	18.07±2.01	17.86±1.92	17.73±1.90	17.85±2.08	**0.006**
TD_3	13.20±1.60	13.28±1.69	13.37±1.32	13.27±1.57	13.07±1.59	12.97±1.82	**0.044**
TD_4	10.18±1.31	10.27±1.29	9.91±1.20	10.31±1.33	10.13±1.29	9.94±1.37	**0.030**
TD_5	7.58±1.01	7.68±1.02	7.36±0.80	7.65±1.00	7.56±1.05	7.38±1.04	0.069
TD_6	6.41±0.95	6.40±0.83	6.23±0.84	6.42±1.00	6.49±0.97	6.36±0.93	**0.031**
TD_7	5.86±0.92	5.91±0.82	5.69±0.68	5.87±0.97	5.88±0.97	5.83±0.85	0.180
TD_8	5.47±0.92	5.60±0.97	5.28±0.69	5.44±0.92	5.51±0.97	5.44±0.84	0.414
TD_9	5.28±1.30	5.09±0.87	4.98±0.81	5.26±1.05	5.33±1.27	5.96±2.58	**0.001**
TD_{10}	5.31±2.19	5.17±1.53	4.76±0.66	5.20±2.02	5.48±2.15	6.56±4.48	**0.039**
**Airway lumen area [mm²]**							
LA_1	283.50±69.09	275.43±68.22	295.92±70.54	283.35±71.83	283.62±65.75	284.40±63.94	0.534
LA_2	147.55±39.22	145.76±40.11	153.31±40.26	148.49±39.90	144.55±37.14	149.87±38.63	0.611
LA_3	68.48±21.08	69.11±22.07	70.50±19.65	69.85±21.07	65.64±20.82	67.90±20.69	0.332
LA_4	36.80±11.32	37.09±11.53	35.60±11.09	37.97±12.05	35.67±10.18	35.89±10.56	0.277
LA_5	20.42±5.40	20.54±5.28	19.90±5.12	21.02±5.82	19.76±4.74	20.14±5.59	0.196
LA_6	15.64±4.17	15.70±3.72	14.97±4.17	15.74±4.25	15.66±4.43	15.77±3.61	0.799
LA_7	13.61±3.69	14.00±3.55	13.06±2.58	13.76±4.00	13.32±3.69	13.72±3.42	0.462
LA_8	12.40±3.86	13.33±4.40	11.59±2.79	12.37±3.67	12.39±4.02	11.85±3.92	0.099
LA_9	12.04±8.53	11.27±3.32	10.45±2.91	11.31±4.25	12.08±6.94*	18.20±22.81	**0.001**
LA_{10}	14.22±25.77	11.52±6.38	9.83±2.73	13.16±22.89	15.18±20.84	30.64±67.91	0.063
**Airway wall percentage [%]**							
WP_1	36.50±4.22	36.78±3.65	36.02±3.99	36.51±4.58	36.74±4.08	35.60±3.98	0.441
WP_2	42.94±5.35	43.04±4.79	42.17±4.27	42.92±6.03	43.47±5.12	42.00±4.48	0.374
WP_3	54.36±6.35	54.39±7.04	53.73±5.40	54.12±6.18	55.24±6.53	53.21±5.80	0.240
WP_4	57.75±6.72	57.74±7.59	57.26±6.20	57.60±6.32	58.42±6.82	56.77±6.87	0.550
WP_5	56.32±7.32	56.51±8.21	55.12±6.22	56.03±7.26	57.25±7.35	55.52±6.47	0.270
WP_6	52.67±8.01	51.68±9.06	52.20±6.97	52.46±8.37	53.84±7.32	52.14±6.90	0.250
WP_7	50.23±8.32	49.43±9.25	48.97±8.38	49.78±7.95	51.62±8.42	50.71±6.92	0.123
WP_8	48.22±8.70	46.90±9.75	47.67±8.30	47.83±8.58	49.09±8.29	50.36±8.35	0.175
WP_9	47.76±9.28	44.80±9.65	46.65±7.76	48.62±9.46	48.36±9.14	49.32±8.59	**0.036**
WP_{10}	46.60±10.37	45.76±11.75	45.47±8.48	47.05±10.66	47.39±9.80	45.84±8.58	0.868

Means and standard deviations for wall thickness (WT), total diameter (TD), lumen area (LA) and wall percentage (WP) for the 1st-10th airway generation and all GOLD grades (Risk COPD, GOLD1, GOLD2, GOLD3, GOLD4).

Supplemental Table 6. Spearman rank order correlations.

	Parenchy. defects	Perfusion defects	Central airway disease	Large airway disease	Small airway disease	Airway score	Global score
**Semiquantitative MRI and quantitative CT**							
Parenchymal disease							
PRM_{fSAD} [%]	0.24	0.39	-0.07	0.13	0.08	0.11	0.35
	**(<0.001)**	**(<0.001)**	(0.105)	**(<0.001)**	(0.081)	(0.015)	**(<0.001)**
PRM_{Emph} [%]	0.61	0.60	-0.08	0.09	0.08	0.07	0.55
	**(<0.001)**	**(<0.001)**	(0.074)	**(0.045)**	(0.082)	(0.131)	**(<0.001)**
$PRM_{Abnormal}$ [%]	0.51	0.56	-0.10	0.010	0.08	0.07	0.50
	**(<0.001)**	**(<0.001)**	**(0.025)**	**(0.024)**	(0.064)	(0.114)	**(<0.001)**
Airway disease							
BE	0.21	0.13	0.02	0.01	0.13	0.03	0.16
	**(<0.001)**	**(0.003)**	(0.633)	(0.903)	**(<0.001)**	(0.548)	**(<0.001)**
$WP_{1\text{-}2}$ [%]	-0.17	-0.17	0.06	0.06	0.08	0.09	-0.12
	**(<0.001)**	**(<0.001)**	(0.130)	(0.118)	**(0.002)**	**(0.031)**	**(0.003)**
$WP_{3\text{-}5}$ [%]	-0.16	-0.15	0.12	0.31	0.04	0.32	0.01
	**(<0.001)**	**(<0.001)**	**(0.004)**	**(<0.001)**	(0.307)	**(<0.001)**	(0.928)
$WP_{6\text{-}10}$ [%]	-0.16	-0.07	0.06	0.20	0.06	0.19	-0.02
	**(<0.001)**	(0.138)	(0.123)	(0.123)	(0.123)	**(<0.001)**	(0.776)
**Semiquantitative MRI and PFT**							
FEV1 [L]	-0.30	-0.34	0.03	-0.07	-0.09	-0.01	-0.27
	**(<0.001)**	**(<0.001)**	(0.513)	(0.071)	**(0.032)**	(0.094)	**(<0.001)**
FEV1/FVC [%]	-0.45	-0.56	0.10	-0.10	-0.06	-0.06	-0.44
	**(<0.001)**	**(<0.001)**	**(0.016)**	**(0.019)**	(0.168)	(0.140)	**(<0.001)**
FEV1pp [%]	-0.40	-0.49	0.02	-0.13	-0.08	-0.121	-0.41
	**(<0.001)**	**(<0.001)**	(0.610)	**(0.002)**	(0.067)	**(0.003)**	**(<0.001)**
T_{LCO} [mmol/min/kPa]	-0.30	-0.35	0.06	0.02	-0.05	0.02	-0.25
	**(<0.001)**	**(<0.001)**	(0.123)	(0.681)	(0.257)	(0.590)	**(<0.001)**

Spearman rank order correlation coefficients were calculated between semiquantitative MRI scores for parenchymal disease (parenchymal and perfusion defect scores) and QCT parametric response mapping (PRM_{fSAD}, PRM_{Emph}, $PRM_{Abnormal}$). In addition, semiquantitative MRI scores for central, large and small airway disease were correlated with QCT airway parameters bronchiectasis index (BE), and wall percentage (WP) pooled for central (airway generation 1-2 ($WP_{1\text{-}2}$)), large (airway generation 3-5 ($WP_{3\text{-}5}$)), and subsegmental (airway generation ($WP_{6\text{-}10}$)) airways. Semiquantitative MRI scores were also correlated with the lung function parameters (forced expiratory volume in 1 s (FEV_1), the ratio of FEV_1 over FVC (FEV_1/FVC), FEV1 percent predicted and transfer factor of the lung for carbon monoxide (T_{LCO}). MRI airway score and MRI global disease score were also correlated. Data re Spearman's ρ with corresponding *P*-value in parentheses.

SUPPLEMENTAL REFERENCES

1 Wielpütz MO, Eichinger M, Wege S et al (2019) Midterm Reproducibility of Chest Magnetic Resonance Imaging in Adults with Clinically Stable Cystic Fibrosis and Chronic Obstructive Pulmonary Disease. Am J Respir Crit Care Med 200:103-107

2 Schiwek M, Triphan SMF, Biederer J et al (2022) Quantification of pulmonary perfusion abnormalities using DCE-MRI in COPD: comparison with quantitative CT and pulmonary function. Eur Radiol 32:1879-1890

3 Eichinger M, Puderbach M, Fink C et al (2006) Contrast-enhanced 3D MRI of lung perfusion in children with cystic fibrosis--initial results. Eur Radiol 16:2147-2152

4 Korosec FR, Frayne R, Grist TM, Mistretta CA (1996) Time-resolved contrast-enhanced 3D MR angiography. Magn Reson Med 36:345-351

5 Triphan SMF, Biederer J, Burmester K et al (2018) Design and application of an MR reference phantom for multicentre lung imaging trials. PLoS One 13:e0199148

6 Konietzke P, Weinheimer O, Wielpütz MO et al (2018) Validation of automated lobe segmentation on paired inspiratory-expiratory chest CT in 8-14 year-old children with cystic fibrosis. PLoS One 13:e0194557

7 Weinheimer O, Achenbach T, Bletz C, Duber C, Kauczor HU, Heussel CP (2008) About objective 3-d analysis of airway geometry in computerized tomography. IEEE Trans Med Imaging 27:64-74

8 Weinheimer O, Achenbach T, Düber C (2009) Fully Automated Extraction of Airways from CT Scans Based on Self-Adapting Region Growing. Second International Workshop on Pulmonary Image Analysis

9 Weinheimer O, Wielpütz M, Konietzke P et al (2017) Fully automated lobe-based airway taper index calculation in a low dose MDCT CF study over 4 time-points. Proc SPIE 10133, Medical Imaging 2017

10 Gould GA, MacNee W, McLean A et al (1988) CT measurements of lung density in life can quantitate distal airspace enlargement--an essential defining feature of human emphysema. Am Rev Respir Dis 137:380-392

11 Wang Z, Gu S, Leader JK et al (2013) Optimal Threshold in CT Quantification of Emphysema. European Radiology 23:975-984

12 Galbán CJ, Han MK, Boes JL et al (2012) Computed tomography-based biomarker provides unique signature for diagnosis of COPD phenotypes and disease progression. Nat Med 18:1711-1715

13 Weinheimer O, Wielpütz MO, Konietzke P et al (2019) Improving pulmonary lobe segmentation on expiratory CTs by using aligned inspiratory CTs. SPIE Medical Imaging 10950

14 Nauck S, Jobst B, von Stackelberg O et al (2024) Phenotyping of COPD with MRI in comparison to same-day CT in a multi-centre trial. European Radiology (in press)

15 Jobst BJ, Triphan SM, Sedlaczek O et al (2015) Functional lung MRI in chronic obstructive pulmonary disease: comparison of T1 mapping, oxygen-enhanced T1 mapping and dynamic contrast enhanced perfusion. PLoS One 10:e0121520
